# Halogen Bonding Involving CO and CS with Carbon as the Electron Donor

**DOI:** 10.3390/molecules22111955

**Published:** 2017-11-12

**Authors:** Janet E. Del Bene, Ibon Alkorta, José Elguero

**Affiliations:** 1 Department of Chemistry, Youngstown State University, Youngstown, OH 44555, USA; 2 Instituto de Química Médica (IQM-CSIC), Juan de la Cierva, 3, E-28006 Madrid, Spain; iqmbe17@iqm.csic.es

**Keywords:** ab initio studies, halogen bonding, structures and binding energies, bonding properties, stretching frequencies, ^13^C chemical shieldings, EOM-CCSD spin–spin coupling constants

## Abstract

MP2/aug’-cc-pVTZ calculations have been carried out to investigate the halogen-bonded complexes formed when CO and CS act as electron-pair donors through C to ClF, ClNC, ClCl, ClOH, ClCN, ClCCH, and ClNH_2_. CO forms only complexes stabilized by traditional halogen bonds, and all ClY molecules form traditional halogen-bonded complexes with SC, except ClF which forms only an ion-pair complex. Ion-pair complexes are also found on the SC:ClNC and SC:ClCl surfaces. SC:ClY complexes stabilized by traditional halogen bonds have greater binding energies than the corresponding OC:ClY complexes. The largest binding energies are found for the ion-pair SC–Cl^+^:^−^Y complexes. The transition structures which connect the complex and the ion pair on SC:ClNC and SC:ClCl potential surfaces provide the barriers for inter-converting these structures. Charge-transfer from the lone pair on C to the σ-hole on Cl is the primary charge-transfer interaction stabilizing OC:ClY and SC:ClY complexes with traditional halogen bonds. A secondary charge-transfer occurs from the lone pairs on Cl to the in-plane and out-of-plane π antibonding orbitals of ClY. This secondary interaction assumes increased importance in the SC:ClNH_2_ complex, and is a factor leading to its unusual structure. C–O and C–S stretching frequencies and ^13^C chemical shieldings increase upon complex formation with ClY molecules. These two spectroscopic properties clearly differentiate between SC:ClY complexes and SC–Cl^+^:^−^Y ion pairs. Spin–spin coupling constants ^1x^J(C–Cl) for OC:ClY complexes increase with decreasing distance. As a function of the C–Cl distance, ^1x^J(C–Cl) and ^1^J(C–Cl) provide a fingerprint of the evolution of the halogen bond from a traditional halogen bond in the complexes, to a chlorine-shared halogen bond in the transition structures, to a covalent bond in the ion pairs.

## 1. Introduction

The hydrogen bond, first described in detail in the classic book of Pimentel [1], has long been the dominant intermolecular interaction in chemistry and biology. However, over the last several years, four new types of intermolecular interactions have received considerable attention. These include halogen bonds [2,3,4] involving Group 17 elements as the acid, chalcogen bonds [5,6,7,8] with Group 16, pnicogen bonds [9,10,11] with Group 15, and tetrel bonds [12,13,14] with Group 14. To emphasize the similarity among these interactions, Desiraju et al. have suggested that they should be considered as arising when a σ-hole [15,16] or a π-hole on an atom E in a molecule from one of these groups interacts with a nucleophilic region such as a pair of nonbonding or π electrons in another, or the same, molecular entity [17].

Legon et al., [18] employed rotational spectroscopy to investigate complexes of CO with Cl_2_ [19], Br_2_ [20], ClF [21], BrCl [22], and ICl [23]. FTIR studies of the complexes of CO with Cl_2_, BrCl, ICl, and IBr were carried out by Romano and Downs [24]. In addition, several computational studies of halogen-bonded complexes in which CO acts as the electron donor have been reported. These include an early investigation of charge transfer in complexes involving dihalogen compounds and CO [25], and a study of the competition between halogen bonds and hydrogen bonds in 1:1 complexes involving CO and hypohalous acids [26]. Studies of the complexes formed between CO and dimers and trimers of dihalogen molecules XY, for X, Y = Cl, Br, have been published [27,28], as well as a study of the difference between standard and counterpoise-corrected optimized geometries of halogen- and hydrogen-bonded complexes [29]. A recent conference on Halogen Bonding highlighted both experimental and theoretical studies of this important intermolecular interaction [30]. Taken together, these two approaches can lead to a better understanding of, and greater insights into, the halogen bond. 

**Scheme 1:**
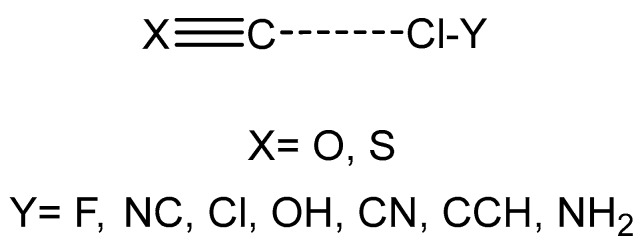
Complexes of CO and CS with ClY molecules.

We have further extended our studies of halogen-bonded complexes to include CO and CS as electron-pair donors through C to the chlorine derivatives ClF, ClNC, ClCl, ClOH, ClCN, ClCCH, and ClNH_2_, which are depicted in Scheme 1. We have searched the XC:ClY potential surfaces not only for complexes stabilized by traditional halogen bonds, but also for those stabilized by chlorine shared or ion-pair bonds [31,32,33,34,35,36,37,38,39]. In this paper, we report and discuss the structures and binding energies of XC:ClY complexes, the energy profile along the intrinsic reaction coordinate for the inter-conversion of the complex and the ion pair through the transition structure, charge-transfer energies for complexes with traditional halogen bonds, bonding data, changes in IR stretching frequencies and ^13^C chemical shieldings upon complex formation, and spin–spin coupling constants across halogen bonds and selected covalent bonds.

## 2. Methods

Searches of the potential surfaces of XC:ClY for X = O and S, and Y = F, NC Cl, OH, CN, CCH, and NH_2_, were carried out for complexes stabilized by C**^…^**Cl halogen bonds, ion pairs with C–Cl covalent bonds, and the transition structures that inter-convert complex and ion pair. These searches were carried out using second-order Møller–Plesset perturbation theory (MP2) [40,41,42,43] with the aug’-cc-pVTZ basis set [44], a basis set derived from the Dunning aug-cc-pVTZ basis set [45,46] by removing diffuse functions from H atoms. Frequencies were computed to establish that complexes and ion pairs correspond to equilibrium structures on their potential surfaces with no imaginary frequencies, and that transition structures have one imaginary frequency along the inter-conversion coordinate. Intrinsic reaction coordinate (IRC) calculations [47,48] were carried out to illustrate the energy profile that connects the two minima through the transition state. Optimization, frequency, and IRC calculations were performed using the Gaussian 09 program [49].

The binding energies of the XC:ClY complexes and ion pairs were computed as the negative of the reaction energy (−ΔE) for the formation of these entities from the corresponding XC and ClY molecules. For consistency, the binding energies of the transition structures were computed in the same way, so that a positive binding energy means that the transition structure is stable relative to the corresponding monomers.

Molecular electrostatic potentials (MEPs) have been evaluated with the DAMQT program [50]. The electron densities of complexes and ion pairs have been analyzed using the Atoms in Molecules (AIM) methodology [51,52,53,54] employing the AIMAll program [55]. The topological analysis of the electron density produces the molecular graph for each complex and ion pair. This graph identifies the location of electron-density features of interest, including the electron-density maxima associated with the various nuclei, and saddle points which correspond to bond critical points (BCPs). The zero-gradient line which connects a BCP with two nuclei is the bond path. The electron density at each intermolecular bond critical point (*ρ*_BCP_), the Laplacian (∆∇^2^*ρ*_BCP_) at that point, and the total energy density (H_BCP_) have been evaluated.

The Natural Bond Orbital (NBO) method [56] has been used to obtain the stabilizing charge-transfer interactions for the OC:ClY and SC:ClY complexes with traditional halogen bonds, using the NBO-6 program [57]. Since MP2 orbitals are nonexistent, charge-transfer interactions have been computed using the B3LYP functional with the aug’-cc-pVTZ basis set at the MP2/aug’-cc-pVTZ complex geometries. This allows for the inclusion of some electron correlation effects.

NMR absolute chemical shieldings have been evaluated at MP2/aug’-cc-pVTZ employing the Gauge-Invariant Atomic Orbital (GIAO) method [58,59] as implemented in the Gaussian-09 program. Coupling constants ^1x^J(C–Cl) and ^1x^J(Cl–A) across halogen bonds, and ^1^J(C–O), ^1^J(C–S), ^1^J(C–Cl), and ^1^J(Cl–A) across covalent bonds, with A being the Y atom directly bonded to Cl in ClY, were evaluated using the equation-of-motion coupled cluster singles and doubles (EOM-CCSD) method in the CI (configuration interaction)-like approximation [60,61], with all electrons correlated. For these calculations, the Ahlrichs [62] qzp basis set was placed on ^13^C, ^15^N, ^17^O, and ^19^F, and the qz2p basis set on ^33^S and ^35^Cl. The Dunning cc-pVDZ basis set was used for ^1^H atoms [62]. The coupling constants were evaluated as the sum of the paramagnetic spin orbit (PSO), diamagnetic spin orbit (DSO), Fermi contact (FC), and spin dipole (SD) terms. The EOM-CCSD calculations were performed using ACES II [63] on the HPC cluster Oakley at the Ohio Supercomputer Center.

## 3. Results and Discussion

### 3.1. CO and CS Monomers

**Figure 1:**
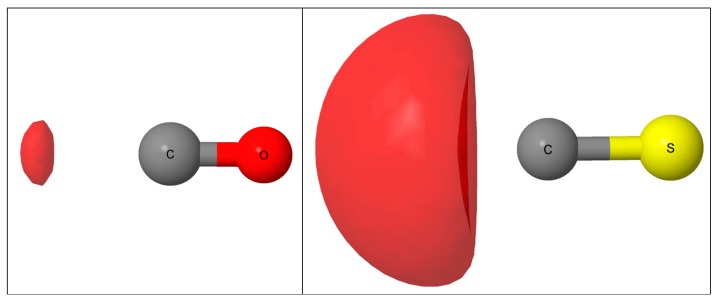
MEPs on the −0.025 au isosurfaces of CO and CS.

The molecules CO and CS may act as electron donors through C to ClY, forming C**^…^**Cl halogen bonds. It is advantageous to examine the molecular electrostatic potentials (MEPs) on the −0.025 au isosurfaces of these two molecules. Figure 1 illustrates these isosurfaces, which are obviously quite different. The MEP minimum at C of CO has a value of −0.028 au, while the MEP minimum at C of CS has a value of −0.061 au and is much more extensive. These results suggest that CS should form stronger halogen bonds.

### 3.2. OC:ClY Complexes

#### 3.2.1. Structures, Binding Energies, Charge-Transfer Energies, and Bonding Properties

**Figure 2:**
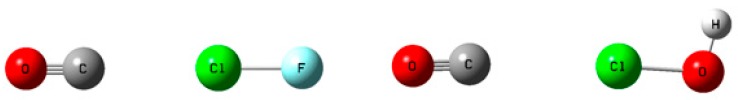
The complexes OC:ClF and OC:ClOH.

Table S1 of the Supplementary Material presents the structures, total energies, and molecular graphs of the OC:ClY complexes. The C–Cl distances and the binding energies of equilibrium OC:ClY complexes are reported in Table 1, and the complexes OC:ClF and OC:ClOH are illustrated in Figure 2. Except for OC:ClOH and OC:ClNH_2_, these complexes have linear structures with *C_∞v_* symmetry. OC:ClOH and OC:ClNH_2_ have C_s_ symmetry, but the O–C–Cl and C–Cl–A angles in both complexes approach 180°, with values of 177° in OC:ClOH, and 173° and 175°, respectively, in OC:ClNH_2_. This linear or nearly linear alignment directs the lone pair on C toward the σ-hole of Cl for the formation of the halogen bond. The C–Cl distances in these complexes vary from 2.662 Å in OC:ClF to 3.288 Å in OC:ClCCH. The gas-phase experimental C–Cl distances in OC:ClF and OC:ClCl are 2.770 Å and 3.092 Å, respectively [19,21], and are longer than the computed distances. This is as expected, since the experimental distances are R_o_ values whereas the computed distances are R_e_.

**Figure 3:**
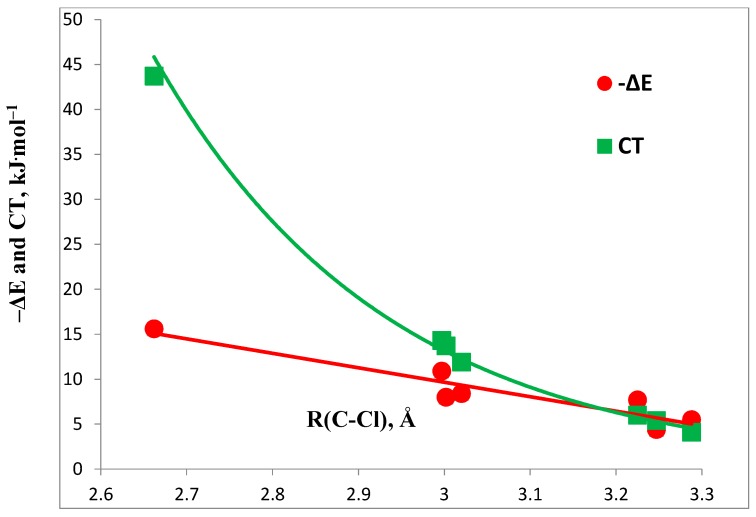
Binding energies and charge-transfer energies versus the C–Cl distance in complexes OC:ClY.

The binding energies of these complexes vary from 4.4 kJ**^.^**mol^−1^ for OC:ClNH_2_ to 15.6 kJ**^.^**mol^−1^ for OC:ClF. Figure 3 illustrates the dependence of the binding energies on the C–Cl distance. The second-order trend line has a correlation coefficient of 0.891. Both the binding energies of these complexes and the C–Cl distances indicate that these complexes are stabilized by traditional halogen bonds, although the C–Cl distance in ClF suggests that the C**^…^**Cl bond in this complex has some chlorine-shared character [31].

Table 1 also reports the charge-transfer energies in these complexes. The primary stabilizing charge-transfer occurs from the lone pair on C to the σ antibonding Cl–A orbital. Because the OC:ClF complex has the strongest halogen bond and shortest intermolecular distance among the OC:ClX complexes, it also has the largest charge-transfer energy of 44 kJ**^.^**mol^−1^. In the remaining complexes, the charge-transfer energies are between 4 and 14 kJ**^.^**mol^−1^. The exponential trendline in Figure 3 for the charge-transfer energies versus the C–Cl distance has a correlation coefficient of 0.993. It is important to note that in these complexes there is a secondary charge-transfer interaction involving electron donation by the lone pairs on Cl to the in-plane and out-of-plane π* orbitals of CO. These charge-transfer energies are significantly less than the primary charge-transfer energy in each complex. Their sum is 10.3 kJ**^.^**mol^−1^ for OC:ClF, and varies between 0.9 and 2.8 kJ**^.^**mol^−1^ for the remaining complexes. It should be noted, however, that charge-transfer energies are not observable properties, and that NBO values are usually too high, given that the primary charge-transfer energy of a complex may be even greater than its binding energy. Nevertheless, they do provide useful insights about charge flow in complexes, and correlate with the intermolecular distance across the halogen bond. 

The results of the AIM analyses for the OC:ClY complexes are reported in Table S2 of the Supplementary Material. Values of electron densities at bond critical points (*ρ*_BCP_), Laplacians (∇^2^*ρ*_BCP_) at those points, and the total energy densities (H_BCP_) are small and positive: *ρ*_BCP_ does not exceed 0.025 au, ∇^2^*ρ*_BCP_ varies between 0.028 and 0.084 au, and H_BCP_ values are 0.002 au except for OC:ClF, in which case it is 0.000 au. The values of *ρ*_BCP_ and ∇^2^*ρ*_BCP_ are plotted against the C–Cl distance in Figure S1. The exponential trend lines have correlation coefficients of 0.999 and 0.994, respectively [12,64,65,66,67,68,69,70,71,72,73]. Thus, the AIM analyses also indicate that the halogen bonds in these complexes are traditional halogen bonds, with that bond in OC:ClF having some chlorine-shared character.

#### 3.2.2. Spectroscopic Properties

Complex formation leads to increases in both the C–O stretching frequency and the ^13^C chemical shielding relative to the CO monomer, and these increases are reported in Table 1. The increase in the C–O stretching frequency varies between 2.1 and 16.4 cm^−1^, and the increase in the ^13^C chemical shielding varies between 0.9 and 4.2 ppm. Both the increase in the C–O stretching frequency and the increase in the ^13^C chemical shielding correlate linearly with the binding energy, with correlation coefficients of 0.939 and 0.950, respectively.

**Figure 4:**
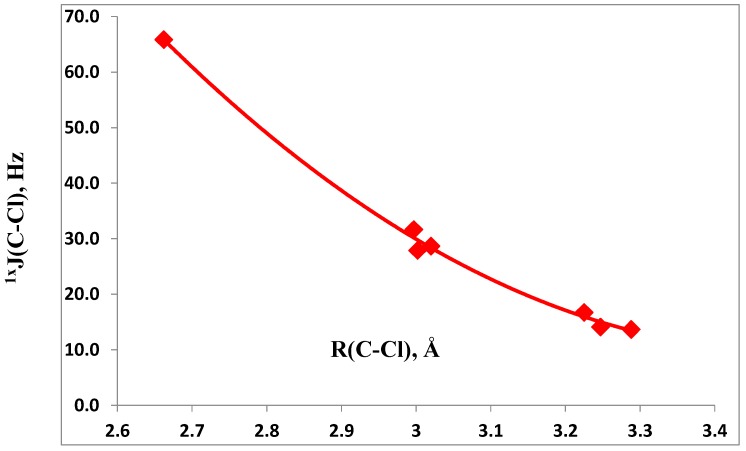
^1x^J(C–Cl) versus the C–Cl distance for OC:ClY complexes.

The components of spin–spin coupling constants ^1x^J(C–Cl) across the halogen bond and ^1^J(C–O) for the covalent C–O bond are reported in Table S3 of the Supplementary Material. As evident from these data, the FC terms are excellent approximations to ^1x^J(C–Cl), but they are not good approximations to ^1^J(C–O) due to contributions from the PSO and SD terms. Total coupling constants ^1x^J(C–Cl), which are also reported in Table 1, vary from 13.7 Hz in OC:ClCCH to 65.9 Hz in OC:ClF. Figure 4 provides a plot of ^1x^J(C–Cl) versus the C–Cl distance. ^1x^J(C–Cl) increases as the C–Cl distance decreases, and the second-order trend line illustrating this dependence has a correlation coefficient of 0.996. This pattern is typical for complexes stabilized by traditional halogen bonds [37,39,74,75]. Neither ^1^J(C–O) nor the C–O distance is very sensitive to complex formation. ^1^J(C–O) varies between 20.2 and 20.8 Hz for C–O distances between 1.137 and 1.139 Å. For the CO monomer, ^1^J(C–O) is 20.7 Hz at a C–O distance of 1.139 Å.

### 3.3. SC:ClY Complexes

#### 3.3.1. Structures, Binding Energies, Charge-Transfer Energies, and Bonding Properties

Unlike the OC:ClY complexes, SC:ClY complexes exhibit some interesting variations in their structures and binding energies. Most obvious is the existence of complexes stabilized by ion-pair halogen bonds subsequent to Cl transfer to SC. On the SC:ClF surface, only one equilibrium structure is found which corresponds to a complex stabilized by an ion-pair halogen bond; both traditional and ion-pair halogen-bonded complexes exist on the SC:ClNC and SC:ClCl surfaces; and only complexes with traditional halogen bonds are found on the remaining surfaces.

**Figure 5:**
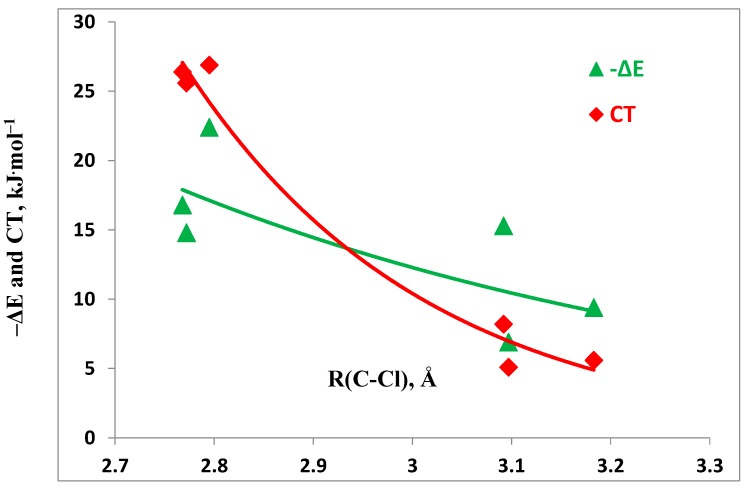
Binding energies and charge-transfer energies versus the C-Cl distance for Complexes SC:ClY.

The structures, total energies, and molecular graphs of complexes stabilized by traditional halogen bonds are reported in Table S4. Their binding energies, intermolecular distances, and symmetries are reported in Table 2. The binding energies range from 6.9 kJ**^.^**mol^−1^ for SC:ClNH_2_ to 22.4 kJ**^.^**mol^−1^ for SC:ClNC. For fixed ClY, the binding energy of SC:ClY is greater than the binding energy of OC:ClY, since CS has the greater MEP minimum at C and is the better electron-pair donor. The C–Cl distance varies from 2.77 Å in SC:ClCl and SC:ClOH to 3.18 Å in SC:ClCCH. Figure 5 provides a plot of the binding energies versus the C–Cl distance, which shows that the correlation between these two variables is poor. The exponential trendline has a correlation coefficient of only 0.535. Closer examination of Table 2 and Figure 5 indicates that the lack of correlation may be due primarily to the large binding energy of SC:ClCN of 15.3 kJ**^.^**mol^−1^ at a long C–Cl distance of 3.092 Å. For comparison, SC:ClCl and SC:ClOH have binding energies of 16.8 and 14.8 kJ**^.^**mol^−1^ at C–Cl distances of 2.77 Å, while SC:ClCCH and SC:ClNH_2_ have binding energies of 9.4 and 6.9 kJ**^.^**mol^−1^ at C–Cl distances of 3.18 and 3.10 Å, respectively. Thus, the binding energy of SC:ClCN is anomalously high at a long C–Cl distance, but we have found no explanation for this observation.

**Figure 6:**
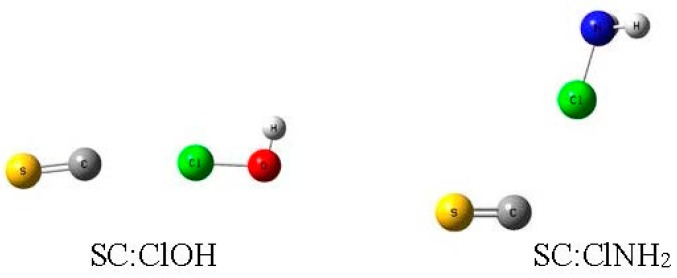
Complexes SC:ClOH and SC:ClNH_2_.

The two complexes that have C_s_ symmetry are SC:ClOH and SC:ClNH_2_, which are illustrated in Figure 6. The structure of SC:ClOH is consistent with the geometry needed for halogen-bond formation, with S–C–Cl and C–Cl–O angles of 174° and 177°, respectively. This nearly linear S–C–Cl–O arrangement facilitates electron donation by C to the σ-hole of Cl. However, this is not the geometry of SC:ClNH_2_. In this complex, the S–C–Cl angle is 122°, and the C–Cl–N angle is 166°. Why does this complex have such a unique structure?

To answer this question, it is advantageous to examine the charge-transfer interactions for the SC:ClY complexes that are reported in Table 2. With one exception, the dominant charge transfer occurs from the carbon lone pair to the σ-hole on Cl. The charge-transfer energies vary from 5.1 to 26.9 kJ**^.^**mol^−1^, and Figure 5 illustrates their exponential dependence on the C–Cl distance, with a correlation coefficient of 0.954. It should be noted that the SC:ClCN complex has a charge-transfer energy which is consistent with its C–Cl distance. However, there is another charge-transfer interaction, namely, back donation of charge from ClY to SC. For these, the charge-transfer energies are the sums of individual charge-transfers from the three lone pairs on Cl to the in-plane and out-of-plane π* orbitals of SC, and are reported in Table 2. Except for SC:ClCCH, these energies are significantly greater than the charge-transfer energies for the corresponding OC:ClY complexes. Nevertheless, in all complexes except SC:ClNH_2_, the Cl_lp_ → π*C–S charge-transfer is a secondary interaction. 

In the SC:ClNH_2_ complex, the sum of the Cl_lp_ → π*C–S charge transfer energies is 6.9 kJ**^.^**mol^−1^. This charge-transfer energy is 1.8 kJ**^.^**mol^−1^ greater than the C_lp_ → σ*Cl–A energy, and is therefore the dominant charge-transfer interaction in this complex. Figure S2 of the Supplementary Material illustrates the orbital interactions associated with the C_lp_ → σ*Cl–A charge-transfer, and the four orbital interactions that contribute to the total Cl_lp_ → π*C–S charge-transfer. The charge-transfer interactions between Cl and the CS π system assume increased importance in the stabilization of this complex, and are linked to its unusual structure which facilitates electron donation by ClNH_2_.

Ion-pair complexes which form subsequent to Cl transfer to C have been found on the SC:ClF, SC:ClCl, and SC:ClNC potential surfaces. Their structures, total energies, and molecular graphs are reported in Table S5, and their binding energies and C–Cl and Cl–A distances are reported in Table 3. These ion-pair complexes have charge-assisted halogen bonds. SC–Cl^+^:^−^F has a binding energy of 114.7 kJ**^.^**mol^−1^ at a C–Cl distance of 1.613 Å. The SC–Cl^+^:^−^Cl ion pair has a binding energy of 47.7 kJ**^.^**mol^−1^ at a C–Cl distance of 1.619 Å, and is significantly more stable than the SC:ClCl complex with a traditional halogen bond which has a binding energy of 16.8 kJ**^.^**mol^−1^. The SC–Cl^+^:^−^NC ion pair has a binding energy of 23.3 kJ**^.^**mol^−1^ at a C–Cl distance of 1.600 Å. The ion-pair complex is only 0.9 kJ**^.^**mol^−1^ more stable than the corresponding complex with a traditional halogen bond.

Since both traditional and ion-pair complexes exist on the SC:ClCl and SC:ClNC surfaces, there is a transition structure which inter-converts them. The SC:ClCl transition structure is bound relative to the corresponding monomers, with a binding energy of 6.6 kJ**^.^**mol^−1^. In contrast, the SC:ClNC transition structure is unbound, with a binding energy of −10.9 kJ**^.^**mol^−1^. On the SC:ClCl surface, the barrier for the reaction complex → ion pair is 10.2 kJ**^.^**mol^−1^, while the barrier for the reverse reaction ion pair → complex is 41.1 kJ**^.^**mol^−1^. The much greater barrier for the reverse reaction arises from the significantly greater binding energy of the ion pair. In contrast, the barriers to the forward and reverse reactions on the SC:ClNC surface are similar at 33.3 and 34.2 kJ**^.^**mol^−1^, respectively, since the binding energies of the complex and ion pair are similar. Figure S3 of the Supplementary Material presents plots of the binding energies along the intrinsic reaction coordinates for these two systems. 

The results of the AIM analyses for the SC:ClY complexes and SC–Cl^+^:^−^Y ion pairs are reported in Table S6 of the Supplementary Material. For a fixed ClY, the electron density at the Cl–Y bond critical point and the Laplacian at that point are more positive in the SC:ClY complex compared with the corresponding OC:ClY complex. This indicates that while the C**^…^**Cl bond is still a traditional halogen bond, it has increased chlorine-shared character in the SC:ClY complexes, consistent with their structural and energetic properties. The energy densities remain small with values of 0.001 and 0.002 au. However, the AIM description of the ion pairs is quite different. The electron densities at C–Cl bond critical points in the ion pairs increase by more than an order of magnitude compared with the complexes with traditional halogen bonds. For the ion pairs, the Laplacians are negative with values between −0.39 and −0.45 au, and the energy densities are also negative with values of about −0.25 au. These data are indicative of the presence of C–Cl covalent bonds in the cations.

#### 3.3.2. Spectroscopic Properties

Table 4 presents the increases in the S–C stretching frequencies and the ^13^C chemical shieldings upon complex formation. The increase in the S–C stretching frequency in complexes with traditional halogen bonds ranges from 2 cm^−1^ in the SC:ClNH_2_ complex to 23 cm^−1^ in SC:ClNC. The increase in the ^13^C chemical shielding lies between 4 and 21 ppm in these same complexes. Both of these variables correlate linearly with the binding energies, with correlation coefficients of 0.990 and 0.939, respectively. The changes in these properties suggest that the C–S stretching frequency and the NMR ^13^C chemical shift should be useful for the experimental characterization of these complexes.

As anticipated, the increases in the C–S stretching frequencies and the ^13^C chemical shieldings are greater in the ion-pair complexes. The increase in the stretching frequencies ranges from 128 to 217 cm^−1^, while the increase in the chemical shielding is between 162 and 192 ppm. Thus, these two spectroscopic properties clearly differentiate between SC:ClY complexes with traditional halogen bonds and SC–Cl^+^:^−^Y ion pairs.

The final spectroscopic properties of interest are the spin–spin coupling constants, including ^1x^J(C–Cl) for coupling across the halogen bond in SC:ClY complexes and transition structures; ^1x^J(Cl–A) for coupling across the new halogen bond in the ion-pair complexes; ^1^J(C–Cl) for the cation in the ion-pair complexes; ^1^J(Cl–A) for the monomers ClF, ClCl, and ClNC; and ^1^J(S–C). The components of these coupling constants are reported in Tables S7 and S8 of the Supplementary Material. The FC term is an excellent approximation to ^1x^J(C–Cl) for complexes with traditional halogen bonds, and a good approximation to this coupling constant in the transition structures. The FC term is not a good approximation to ^1^J(C–Cl) in the ion-pair complexes, ^1x^J(Cl–A) across the new halogen bond in the ion pairs, ^1^J(S–C), or ^1^J(Cl–A) in the monomers ClF, ClCl, and ClNC.

**Figure 7:**
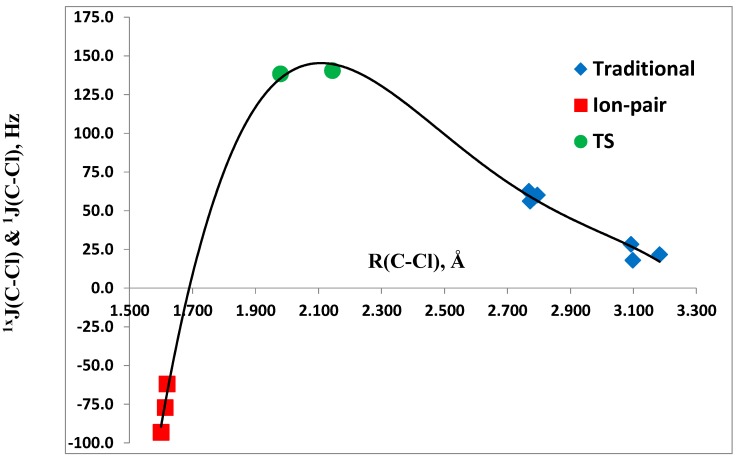
^1x^J(C–Cl) for complexes with traditional halogen bonds and transition structures with chlorine-shared halogen bonds, and ^1^J(C–Cl) for ion-pair complexes versus the C–Cl distance.

Perhaps the most interesting of the coupling constants is ^1x^J(C–Cl) in the complexes and transition structures, which becomes ^1^J(C–Cl) in the ion pairs. These coupling constants provide a fingerprint of the evolution of the halogen bond from a traditional halogen bond in the complexes, to a chlorine-shared halogen bond in the transition structures, and then to a covalent C–Cl bond in the ion pairs, as evident from the plot of Figure 7. At the longest C–Cl distances between 3.09 and 3.18 Å are the complexes SC:ClCN, SC:ClCCH, and SC:ClNH_2_ with ^1x^J(C–Cl) values between 18 and 28 Hz. As the C–Cl distance shortens to between 2.77 and 2.80 Å, ^1x^J(C–Cl) increases to between 53 and 63 Hz in the complexes SC:ClNC, SC:ClCl, and SC:ClOH. These complexes are stabilized by traditional halogen bonds with some chlorine-shared character. The transition structures SC:ClNC and SC:ClCl have chlorine-shared halogen bonds at C–Cl distances of 1.97 and 2.11 Å, and ^1x^J(C–Cl) values of 138 and 141 Hz, respectively. It is in this interval that the trendline curvature changes as chlorine-shared halogen bonds begin to approach ion-pair bonds. At the shortest distances, the coupling constants for the ion pairs change sign with ^1^J(C–Cl) equal to −77, −62, and −93 Hz at C–Cl distances of 1.61, 1.62, and 1.60 Å for the ion pairs SC–Cl^+^:^−^F, SC–Cl^+^:^−^Cl, and SC–Cl^+^:^−^NC, respectively. The differences in the values of these three coupling constants may be attributed to the presence of the different Y^−^ ions.

Coupling constants ^1x^J(Cl–F), ^1x^J(Cl–Cl), ^1x^J(Cl–N) across the new Cl**^…^**A halogen bonds and the Cl–A distances in the ion pairs are reported in Table 4. These distances are much longer than they are in the corresponding ClY monomers since what was a covalent Cl–A bond in the ClY molecule becomes a Cl**^…^**A halogen bond in the ion pair. The increase in the Cl–A distance in the ion pairs leads to a dramatic decrease in ^1x^J(Cl–A), from 798 Hz in ClF to 449 Hz in SC–Cl^+^:^−^F; from 100 Hz in ClCl to 51 Hz in SC–Cl^+^:^−^Cl; and from 34 Hz in ClNC to −49 Hz in SC–Cl^+^:^−^NC.

**Figure 8:**
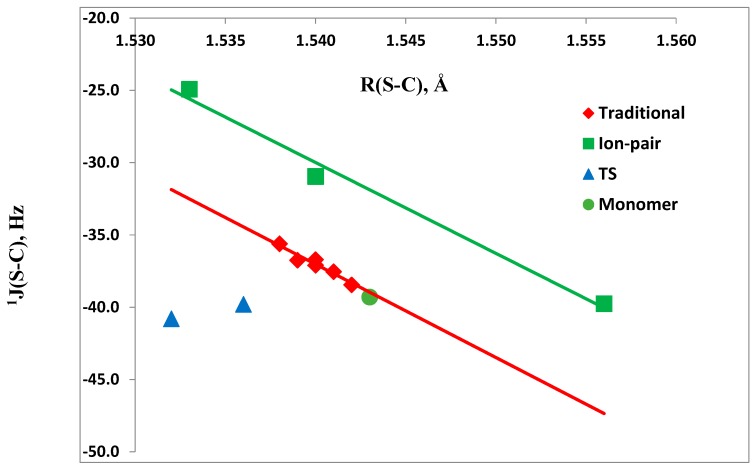
^1^J(S–C) versus the S–C distance for SC:ClY complexes, transition structures, and ion pairs, and the CS monomer.

Table 4 also provides the S–C distances and ^1^J(S–C) values for complexes and ion pairs. Unlike ^1^J(O–C) for the OC:ClY complexes, ^1^J(S–C) is sensitive to the nature of the halogen bond, as can be seen in Figure 8. Although the S–C distance varies by only 0.004 Å and ^1^J(S–C) varies by only 2.8 Hz in the SC:ClY complexes, a linear correlation is found between these two variables, with a correlation coefficient of 0.933. In the three ion-pair complexes, the S–C distance varies by 0.023 Å while ^1^J(S–C) varies by 14.9 Hz. The linear trendline which shows the relationship between ^1^J(S–C) and the S–C distance has a correlation coefficient of 0.986. The trendlines for the complexes and ion pairs are nearly parallel to each other, with the trendline for the ion pairs lying above the trendline for the complexes. The SC:ClNC and SC:ClCl transition structures, which are represented by triangles in Figure 8, have short S–C distances but ^1^J(S–C) values that are similar to the monomer value.

## 4. Conclusions

MP2/aug’-cc-pVTZ calculations have been carried out to investigate the halogen-bonded complexes formed when CO and CS act as electron-pair donors to ClF, ClNC, ClCl, ClOH, ClCN, ClCCH, and ClNH_2_. The results of these calculations support the following statements.

1. CO forms complexes with ClY molecules that are stabilized by traditional halogen bonds. SC:ClY complexes stabilized by traditional halogen bonds are found on all surfaces except SC:ClF, where only an ion-pair SC–Cl^+^:^−^F complex exists. Both traditional halogen-bonded and ion-pair complexes are found on the SC:ClNC and SC:ClCl surfaces.
2. The binding energies of the OC:ClY complexes range from 4 to 16 kJ**^.^**mol**^−1^** while the binding energies of the SC:ClY complexes with traditional halogen bonds are between 7 and 22 kJ**^.^**mol**^−1^.** Ion-pair complexes have binding energies between 23 and 115 kJ**^.^**mol**^−1^.**
3. The transition structures which connect the complex and the ion pair on the SC:ClNC and SC:ClCl surfaces provide the barriers for inter-converting these structures. Converting the complex to the ion pair requires 10 kJ**^.^**mol**^−1^** while the reverse reaction requires 41 kJ**^.^**mol**^−1^** on the SC:ClCl surface. The barriers for these two reactions on the SC:ClNC surface are about 33 kJ**^.^**mol**^−1^** since the complex and ion pair have similar binding energies.
4. Charge-transfer from the lone pair on C to the σ-hole on Cl is the primary charge-transfer interaction stabilizing OC:ClY and SC:ClY complexes with traditional halogen bonds. A secondary charge-transfer occurs from the lone pairs on Cl to the in-plane and out-of-plane antibonding π orbitals of ClY. This secondary interaction assumes increased importance in the SC:ClNH_2_ complex, and is a factor leading to its unusual structure.
5. C–O and C–S stretching frequencies and ^13^C chemical shieldings increase upon complex formation with ClY molecules. These two spectroscopic properties clearly differentiate between SC:ClY complexes and SC–Cl^+^:^−^Y ions pairs.
6. Spin–spin coupling constants ^1x^J(C–Cl) for OC:ClY complexes increase with decreasing distance. For SC:ClY systems, ^1x^J(C–Cl) provides a fingerprint of the evolution of the halogen bond as a function of the C–Cl distance, from a traditional halogen bond in complexes, to a chlorine-shared halogen bond in transition structures, to a covalent bond in ion pairs. ^1x^J(Cl–A) is the coupling constant across the new halogen bond in the ion-pair complexes. It is significantly reduced relative to ^1^J(Cl–A) in the corresponding monomers.

## Figures and Tables

**Table 1:** Binding energies (−ΔE) and charge-transfer energies (C_lp_ → σ*Cl–A and Cl_lp_ → π*C–O, kJ**^.^**mol^−1^), C–Cl distances (R, Å), increases in C–O stretching frequencies (δυ, cm^−1^) and ^13^C chemical shieldings (Δδ^13^C, ppm), and spin–spin coupling constants ^1xJ^(C–Cl) (Hz) for OC:ClY complexes.

ClY	−ΔE	R(C–Cl)	C_lp_ → σ*Cl–A ^a^	Cl_lp_ → π*C–O ^b^	δυ	Δ*δ*^13^C	^1x^J(C–Cl)
ClF	15.6	2.662	43.7	10.3	16.4	4.23	65.9
ClNC	10.9	2.997	14.3	2.6	13.8	3.45	31.7
ClCl	8.4	3.020	11.9	2.5	8.0	2.65	28.7
ClOH	8.0	3.002	13.7	2.8	6.8	2.19	27.9
ClCN	7.7	3.225	6.0	1.0	8.1	2.27	16.7
ClCCH	5.5	3.288	4.1	0.9	4.3	1.26	13.7
ClNH_2_	4.4	3.247	5.4	1.2	2.1	0.94	14.1

^a^ A is the Y atom that is directly bonded to Cl; ^b^ Sum of the charge-transfer energies.

**Table 2:** Binding energies (−ΔE) and charge-transfer energies (C_lp_ → σ*Cl–A and Cl_lp_ → π*C–S, kJ**^.^**mol^−1^), C–Cl distances (R, Å), and symmetries of SC:ClY complexes.

SC:ClY, ClY =	−ΔE	R(C–Cl)	C_lp_ → σ*Cl–A ^a^	Cl_lp_ → π*C–S ^b^	Sym
ClNC	22.4	2.795	26.9	14.7	*C_∞v_*
ClCl	16.8	2.768	26.4	16.0	*C_∞v_*
ClCN	15.3	3.092	8.2	5.6	*C_∞v_*
ClOH	14.8	2.772	25.6	16.9	*C_s_*
ClCCH	9.4	3.183	5.6	0.9	*C_∞v_*
ClNH_2_	6.9	3.097	5.1	6.9	*C_s_*

^a^ A is the Y atom directly bonded to Cl; ^b^ Sum of the charge-transfer energies.

**Table 3:** Binding energies (−ΔE), energies of transition structures (-E_TS_), and barriers to the inter-conversion of complex and ion pair (E^‡^_f_, E^‡^_r_, kJ**^.^**mol^−1^), C–Cl and Cl–A distances (R, Å), and symmetries of SC–C^l+:−^Y ion pairs.

Y =	−ΔE	R(C–Cl)	R(Cl–A) ^a^	−E_TS_	R(C–Cl)_TS_	E^‡^_f_^b^	E^‡^_r_^b^	Sym
F^c^	114.7	1.613	1.917 ^d^					*C_∞v_*
Cl	47.7	1.619	2.379 ^e^	6.6	2.114	10.2	41.1	*C_s_*
NC	23.3	1.600	2.209 ^f^	−10.9	1.979	33.3	34.2	*C_s_*

^a^ A is the Y atom directly bonded to Cl in ClY; ^b^ The forward reaction: complex → ion pair; the reverse reaction: ion pair → complex; **^c^** There is no stable SC:ClF complex with a traditional halogen bond on this surface; ^d^ Isolated Cl–F: R(Cl–F) = 1.638 Å; ^e^ Isolated Cl–Cl: R(Cl–Cl) = 1.999 Å; ^f^ Isolated ClNC: R(Cl–N) = 1.624 Å.

**Table 4:** Increases in C–S stretching frequencies (δν, cm^−1^) and ^13^C chemical shieldings (Δδ^13^C, ppm), Cl–A and S–C distances (Å), and coupling constants ^1x^J(C–Cl), ^1x^J(Cl–A), ^1^J(C–Cl) and ^1^J(S–C) (Hz) for SC:ClY systems.

ClY =	Δν ^a^	Δδ^13^C ^b^	^1x^J(C–Cl)			R(S–C) ^c^	^1^J(S–C) ^c^
ClNC	22.5	21.0	60.2			1.538	−35.6
ClCl	15.9	18.6	62.6			1.539	−36.7
ClCN	11.9	12.4	28.3			1.540	−36.7
ClOH	12.8	14.0	53.1			1.540	−37.1
ClCCH	6.0	6.8	21.7			1.541	−37.5
ClNH_2_	2.2	4.2	18.0			1.542	−38.4
**SCCl^+^:^−^Y, Y =**			**^1^J(C–Cl)**	**^1x^J(Cl–A) ^d^**	**R(Cl–A) ^d^**		
F	159.9	186.1	−77.1	449.3 ^e^	1.917	1.540	−31.0
Cl	128.1	162.3	−61.9	50.7 ^f^	2.379	1.533	−24.9
NC	217.3	191.6	−93.1	−48.6 ^g^	2.209	1.556	−39.8

^a^ The C–S stretching frequency in CS is 1297.1 cm^−1^; ^b^ The ^13^C chemical shielding in CS is −169.3 ppm; ^c^ CS monomer: ^1^J(S–C) = −39.3 Hz at R(S–C) = 1.543 Å; SC:ClNC transition structure: ^1^J(S–C) = −40.8 Hz at R(S–C) = 1.532 Å; SC:ClCl transition structure: ^1^J(S–C) = −39.8 Hz at R(S–C) = 1.536Å; ^d^ A is the Y atom directly bonded to Cl in ClY; ^e^ Cl–F: ^1^J(Cl–F) = 798.4 Hz at R = 1.638 Å; ^f^ Cl–Cl: ^1^J(Cl–Cl) = 99.6 Hz at R = 1.999 Å; ^g^ ClNC: ^1^J(Cl–N) = 34.0 Hz at R = 1.634 Å.

## Supplementary Materials

Supplementary Material is available online and contains structures, total energies, and molecular graphs of complexes, transition structures, and ion pairs; bond critical point data for complexes and ion pairs; plots of bond critical point data; plots of the energy variation along the intrinsic reaction coordinates for SC:ClNC and SC:ClCl systems; and components of spin–spin coupling constants.
