# Supplementary material for: Halogen Bonding Involving CO and CS with Carbon as the Electron Donor
Source: Molecules. 2017 Nov 12;22(11):1955. doi: 10.3390/molecules22111955 (PMC6150174; doi:10.3390/molecules22111955)
Supplement: Supplementary file 1 [file molecules-22-01955-s001.pdf]

Supplementary Material for

Halogen Bonding involving CO and CS with Carbon as the Electron Donor

Janet E. Del Bene,[‡] Ibon Alkorta,[§] José Elguero[§]

[‡] Department of Chemistry, Youngstown State University, Youngstown, Ohio 44555 USA

[§] Instituto de Química Médica (IQM-CSIC), Juan de la Cierva, 3, E-28006 Madrid, Spain

Pgs. S2-S4 Table S1. Structures (Å), total energies (au), and molecular graphs of complexes OC:ClY

Pg. S5 Table S2. Bond critical point data (ρ_{BCP}, $\nabla^2\rho_{BCP}$, and H_{BCP}, au) for OC:ClY complexes

Pg. S5 Fig. S1. Plots of the electron densities (ρ_{BCP}) and the Laplacians ($\nabla^2\rho_{BCP}$) (au) at C···Cl bond critical points versus the C-Cl distance (Å) for OC:ClY complexes

Pg. S6 Table S3. Components of ^{1x}J(C-Cl) and ^{1}J(C-O) (Hz) for OC:ClY complexes

Pgs. S7 –S8 Table S4. Structures (Å), total energies (au), and molecular graphs of SC:ClY complexes stabilized by traditional halogen bonds

Pg. S9 Fig. S2. Orbitals involved in charge-transfer interactions in $SC{:}ClNH_2$

Pg. S10 Table S5. Structures (Å), total energies (au), and molecular graphs of ion-pair complexes $SC\text{-}Cl^{+}{:}^{-}Y$

Pg. S11 Fig. S3. Plots along the intrinsic reaction coordinate (IRC) for the inter-conversion of complex and ion-pair on the SC:ClCl and SC:ClNC potential surfaces

Pg. S12 Table S6. Bond critical point data (ρ_{BCP}, $\nabla^2\rho_{BCP}$, and H_{BCP}, au) for SC:ClY complexes and $SCCl^{+}{:}^{-}Y$ ion-pairs

Pg. S13 Table S7. Components of ^{1x}J(C-Cl) for SC:ClY complexes and transition structures, ^{1}J(C-Cl) and ^{1x}J(Cl-A) for $SCCl^{+}{:}^{-}Y$ ion-pairs, and ^{1}J(Cl-A) for monomers ClF, ClCl, and ClNC

Pg. S14 Table S8. Components of ^{1}J(S-C) for complexes, ion-pairs, transition structures, and the SC monomer

Table S1. Structures (Å), total energies (au), and molecular graphs of complexes OC:ClY

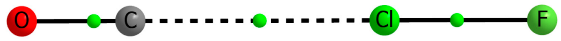	OC:ClF<br>MP2/AUG'-CC-PVTZ= -672.51018972<br>NIMAG= 0<br>C<br>X 1 one<br>O 1 r1 2 nin<br>Cl 1 r2 2 nin 3 tra<br>F 1 r3 2 nin 3 tra<br><br>r1=1.13691199<br>r2=2.66247342<br>r3=4.31429744<br>one=1.<br>nin=90.<br>tra=180.
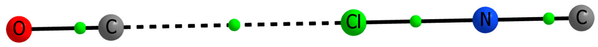	OC:ClNC<br>MP2/AUG'-CC-PVTZ= -665.43891428<br>NIMAG= 0<br>C<br>X 1 one<br>O 1 r1 2 nin<br>Cl 1 r2 2 nin 3 tra<br>N 1 r3 2 nin 3 tra<br>C 1 r4 2 nin 3 tra<br><br>r1=1.13729218<br>r2=2.99675982<br>r3=4.62484783<br>r4=5.81147763<br>one=1.<br>nin=90.<br>tra=180.
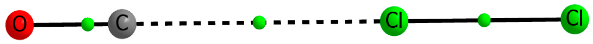	OC:Cl_2<br>MP2/AUG'-CC-PVTZ= -1032.53268157<br>NIMAG= 0<br>C<br>X 1 one<br>O 1 r1 2 nin<br>Cl 1 r2 2 nin 3 tra<br>Cl 1 r3 2 nin 3 tra<br><br>r1=1.13804715<br>r2=3.02005978<br>r3=5.02464523<br>one=1.

<table>
<tr><td></td><td>nin=90.
tra=180.</td></tr>
<tr><td>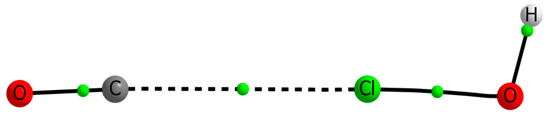
</td><td>OC:ClOH
MP2/AUG’-CC-PVTZ= -648.51622783
NIMAG= 0
C,0.2067799453,0.,0.0160804631
O,1.3430918203,0.,-0.0486658133
Cl,-2.7952238146,0.,0.0545732002
O,-4.4951016769,0.,-0.0068927364
H,-4.7392707386,0.,0.9304234621</td></tr>
<tr><td>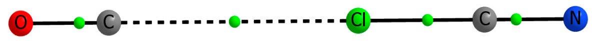
</td><td>OC:ClCN
MP2/AUG’-CC-pVTZ =-665.51482092
NIMAG= 0
C
X 1 one
O 1 r1 2 nin
Cl 1 r2 2 nin 3 tra
C 1 r3 2 nin 3 tra
N 1 r4 2 nin 3 tra

r1=1.13791594
r2=3.22498693
r3=4.85774232
r4=6.03318381
one=1.
nin=90.
tra=180.</td></tr>
<tr><td>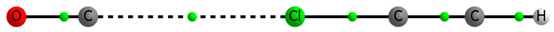
</td><td>OC:ClCCH
MP2/AUG’-CC-PVTZ= -649.41957451
NIMAG= 0
C
X 1 one
O 1 r1 2 nin
Cl 1 r2 2 nin 3 tra
C 1 r3 2 nin 3 tra
C 1 r4 2 nin 3 tra
H 1 r5 2 nin 3 tra

r1=1.13843245
r2=3.2878752
r3=4.92689611
r4=6.14098485
r5=7.20244288
one=1.
nin=90.</td></tr>
</table>

	tra=180.
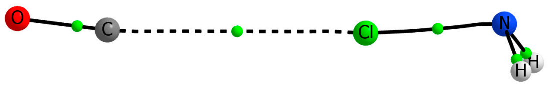 	OC:ClNH$_2$<br>MP2/AUG'-CC-PVTZ= -628.67445117<br>NIMAG= 0<br>C,0.4140444106,0.,0.0472411091<br>O,1.5445254707,0.,-0.0897721001<br>Cl,-2.8332989477,0.,0.0669332624<br>N,-4.5763675504,0.,-0.0603334621<br>H,-4.8636443746,0.810114332,0.4832273376<br>H,-4.8636443746,-0.810114332,0.4832273376

Table S2. Bond critical point data (ρ_{BCP}, $\nabla^2\rho_{BCP}$, and H_{BCP}, au) for OC:ClY complexes

ClY	ρ_{BCP}	$\nabla^2\rho_{BCP}$	H_{BCP}
ClF	0.024	0.084	0.000
ClNC	0.012	0.047	0.002
ClCl	0.012	0.046	0.002
ClOH	0.012	0.047	0.002
ClCN	0.008	0.032	0.002
ClCCH	0.007	0.028	0.002
$ClNH_2$	0.007	0.030	0.002

Fig. S1. Plots of the electron densities (ρ_{BCP}) and the Laplacians ($\nabla^2\rho_{BCP}$) (au) at C···Cl bond critical points versus the C-Cl distance (Å) for OC:ClY complexes

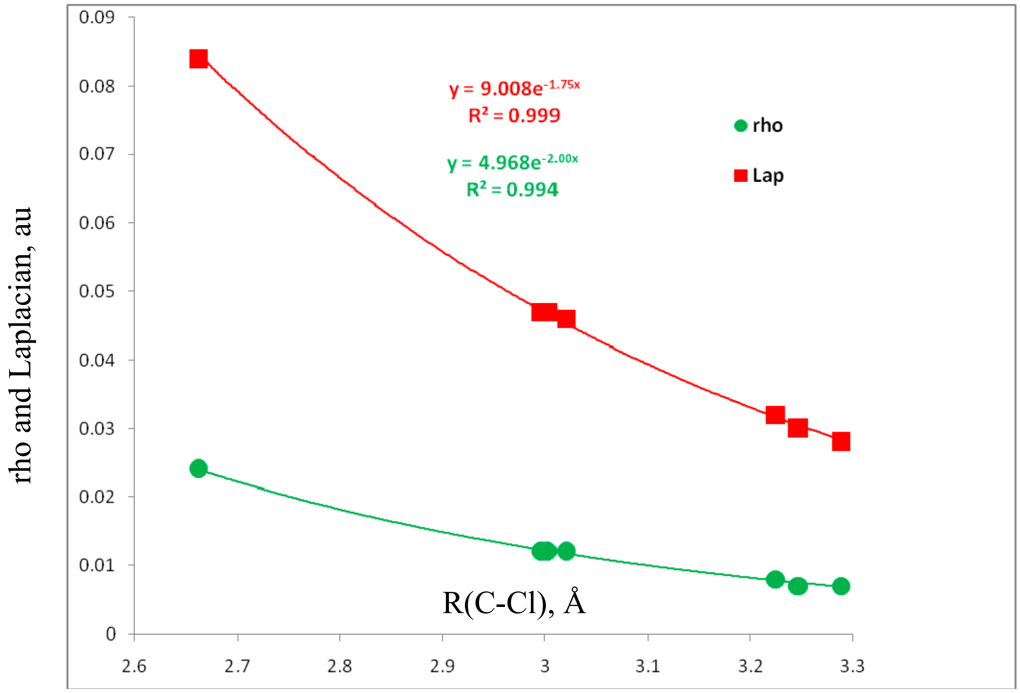

Table S3. Components of ^{1x}J(C-Cl) and ^1J(C-O) (Hz) for OC:ClY complexes

	PSO	DSO	FC	SD	^{1x}J(C-Cl)
OC:ClF	0.1	0.0	66.0	-0.3	65.9
OC:ClNC	0.1	0.0	31.6	0.0	31.7
OC:ClCl	0.2	0.0	28.5	-0.1	28.7
OC:ClOH	0.2	0.0	27.8	-0.1	27.9
OC:ClCN	0.0	0.0	16.7	0.0	16.7
OC:ClCCH	0.0	0.0	13.6	0.0	13.7
OC:ClNH$_2$	0.1	0.0	14.0	0.0	14.1

	PSO	DSO	FC	SD	^1J(C-O)
OC:ClF	12.8	0.1	13.1	-5.1	20.8
OC:ClNC	13.1	0.1	12.0	-5.0	20.2
OC:ClCl	13.3	0.1	12.0	-4.9	20.4
OC:ClOH	13.3	0.1	11.9	-4.9	20.5
OC:ClCN	13.3	0.1	11.9	-4.9	20.3
OC:ClCCH	13.4	0.1	11.8	-4.8	20.4
OC:ClNH2	13.5	0.1	11.7	-4.7	20.5
CO	13.6	0.1	11.8	-4.8	20.7

Table S4. Structures (Å), total energies (au), and molecular graphs of SC:ClY complexes stabilized by traditional halogen bonds

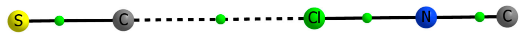	SC:ClNC<br>MP2/AUG'-CC-PVTZ= -987.96243507<br>NIMAG= 0<br>C<br>X,1,one<br>S,1,r1,2,nin<br>Cl,1,r2,2,nin,3,tra,0<br>N,1,r3,2,nin,3,tra,0<br>C,1,r4,2,nin,3,tra,0<br><br>r1=1.53756654<br>r2=2.79539624<br>r3=4.43342209<br>r4=5.61980474<br>one=1.<br>nin=90.<br>tra=180.
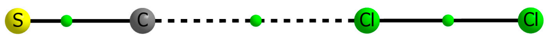	SC:Cl_2<br>MP2/AUG'-CC-PVTZ= -<br>1355.05505377 NIMAG= 0<br>C<br>X,1,one<br>S,1,r1,2,nin<br>Cl,1,r2,2,nin,3,tra,0<br>Cl,1,r3,2,nin,3,tra,0<br><br>r1=1.53893474<br>r2=2.76753341<br>r3=4.78663423<br>one=1.<br>nin=90.<br>tra=180.
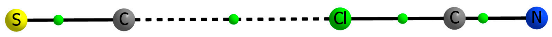	SC:ClCN<br>MP2/AUG'-CC-PVTZ= -988.03689403<br>NIMAG= 0<br>C<br>X,1,one<br>S,1,r1,2,nin<br>Cl,1,r2,2,nin,3,tra,0<br>C,1,r3,2,nin,3,tra,0<br>N,1,r4,2,nin,3,tra,0<br><br>r1=1.53989099<br>r2=3.09180632

	r3=4.72583381<br>r4=5.90154998<br>one=1.<br>nin=90.<br>tra=180.
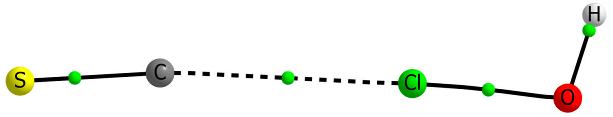	SC:ClOH<br>MP2/AUG’-CC-PVTZ= -971.03799286<br>NIMAG= 0<br>C,-0.0397056027,0.,0.0788470096<br>S,1.4930490506,0.,-0.0694526256<br>Cl,-2.8117144943,0.,0.0656498667<br>O,-4.5206641948,0.,-0.0310402195<br>H,-4.7809401233,0.,0.9015145444
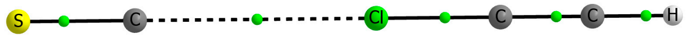	SC:ClCCH<br>MP2/AUG’-CC-PVTZ= -971.94024694<br>NIMAG= 0<br>C<br>X,1,one<br>S,1,r1,2,nin<br>Cl,1,r2,2,nin,3,tra,0<br>C,1,r3,2,nin,3,tra,0<br>C,1,r4,2,nin,3,tra,0<br>H,1,r5,2,nin,3,tra,0<br><br>r1=1.54143742<br>r2=3.18301357<br>r3=4.82282167<br>r4=6.03751802<br>r5=7.09884161<br>one=1.<br>nin=90.<br>tra=180.
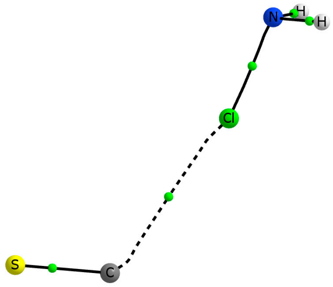	SC:ClNH$_2$<br>MP2/AUG’-CC-PVTZ= -951.19457076<br>NIMAG= 0<br>C,-0.80790239,-1.61348652,0.<br>S,0.31676247,-2.66883094,0.<br>Cl,-0.23290641,1.43016097,0.<br>N,0.50617009,3.0164891,0.<br>H,0.09706841,3.47723698,0.80934564<br>H,0.09706841,3.47723698,-0.80934564

Fig. S2. Orbitals involved in charge-transfer interactions in SC:ClNH$_2$[a,b]

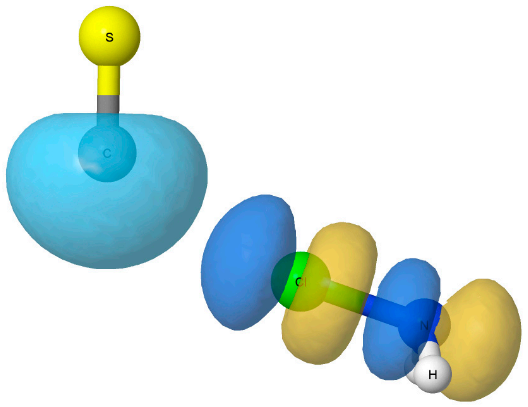

a) The Cl$_{lp}$→σ*Cl-N charge-transfer. The charge-transfer energy is 5.1 kJ/mol.

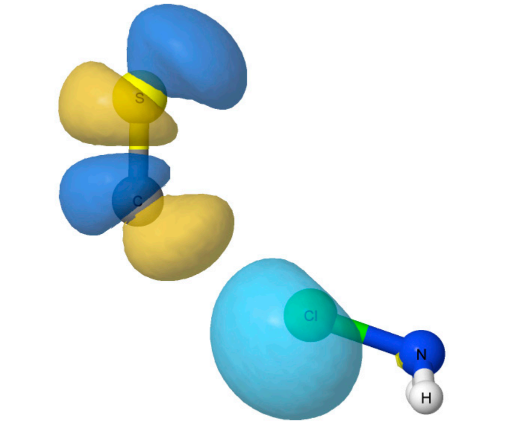	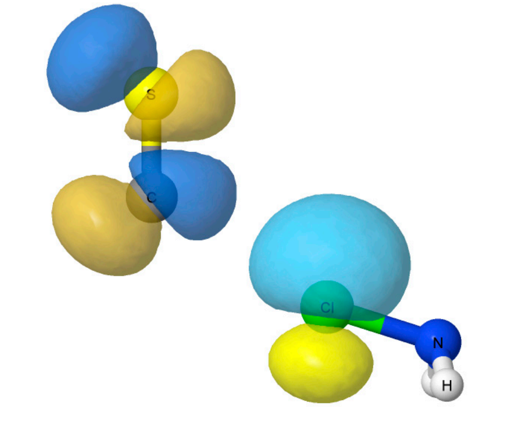
2.6 kJ/mol	1.4 kJ/mol
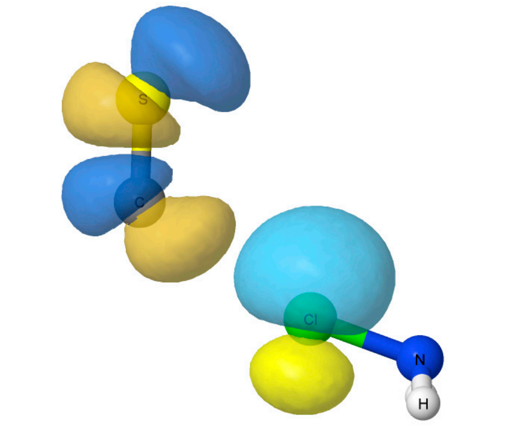	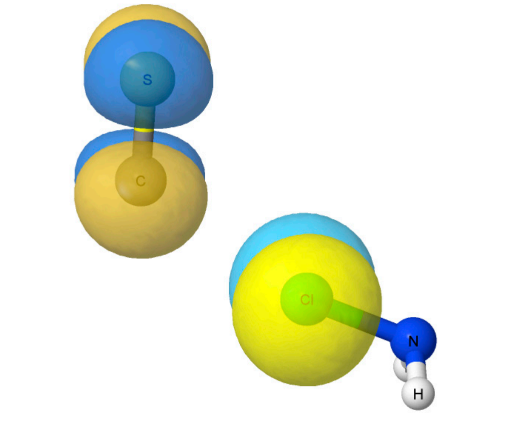
2.0 kJ/mol	0.9 kJ/mol

b) The Cl$_{lp}$→π*C-S back donations and the corresponding charge-transfer energies

Table S5. Structures (Å), total energies (au), and molecular graphs of
ion-pair complexes SC-Cl^{+}:$^{-}$Y

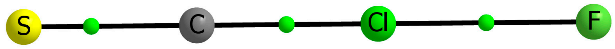	SC-Cl^{+}:$^{-}$F<br>MP2/AUG'-CC-PVTZ= -995.06710205<br>NIMAG= 0<br>C<br>X,1,one<br>S,1,r1,2,nin<br>Cl,1,r2,2,nin,3,tra,0<br>F,1,r3,2,nin,3,tra,0<br><br>r1=1.53964055<br>r2=1.61335012<br>r3=3.53034882<br>one=1.<br>nin=90.<br>tra=180.
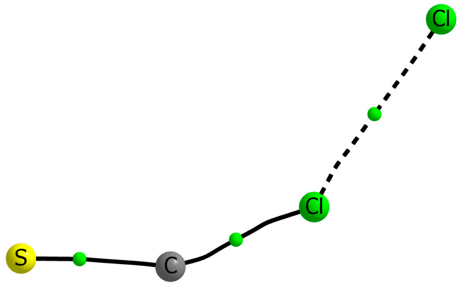	SC-Cl^{+}:$^{-}$Cl<br>MP2/AUG'-CC-PVTZ= -1355.06682708<br>NIMAG= 0<br>C,0.0286935074,1.3216878202,0.<br>S,-1.1592774942,2.326245182,0.<br>Cl,0.6801493973,-0.1601543928,0.<br>Cl,0.2736085895,-2.5040686094,0.
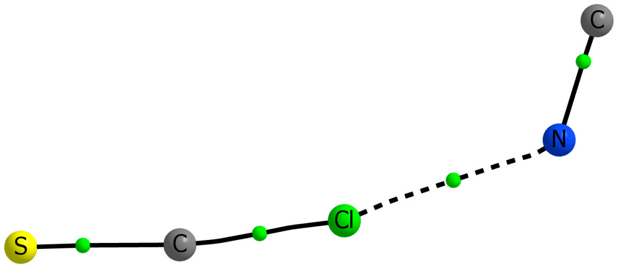	SC-Cl^{+}:$^{-}$NC<br>MP2/AUG'-CC-PVTZ= -987.96278985<br>NIMAG= 0<br>C,-0.2675920233,-1.0282157739,0.<br>S,-0.0301064942,-2.5424570055,0.<br>Cl,-0.3110190858,0.5710337885,0.<br>N,0.0951976604,2.7419744786,0.<br>C,1.1535289428,3.2975325123,0.

Fig. S3. Plots along the intrinsic reaction coordinate (IRC) for the inter-conversion of complex and ion-pair on the SC:ClCl and SC:ClNC potential surfaces

SC:ClCl

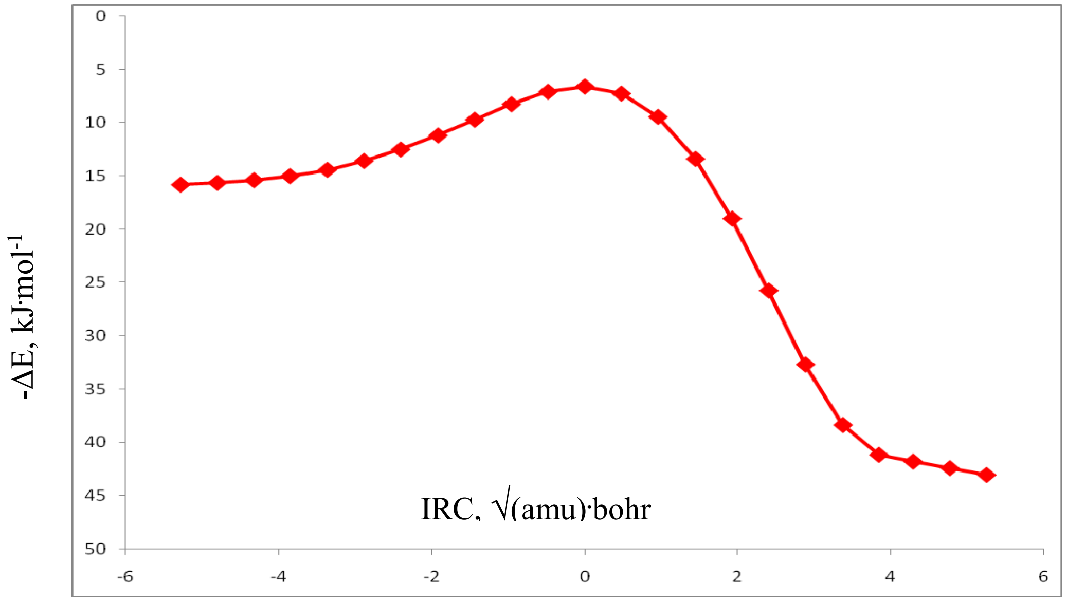

SC:ClNC

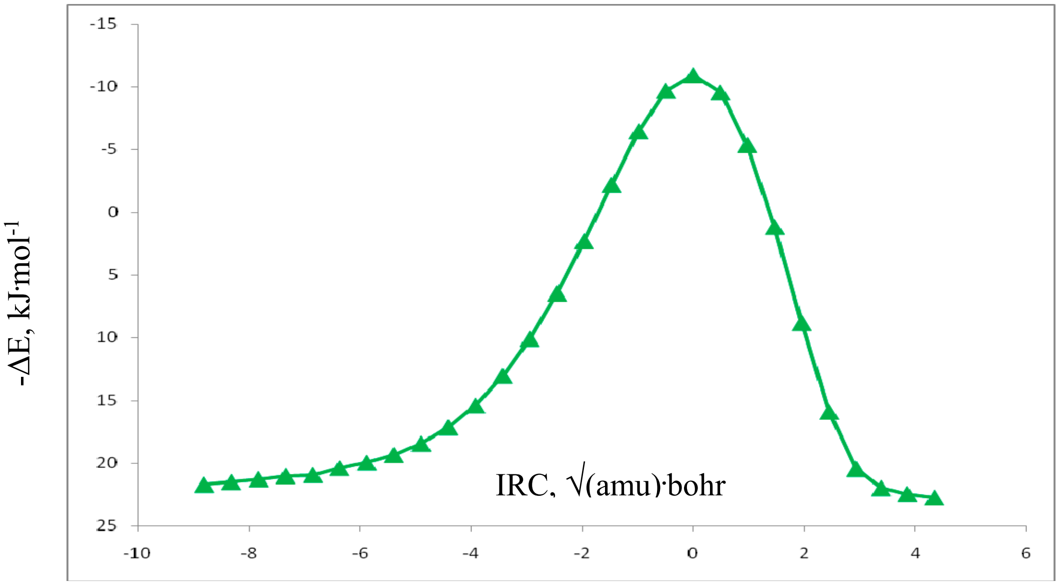

Table S6. Bond critical point data (ρ_{BCP}, $\nabla^2\rho_{BCP}$, and H_{BCP}, au) for SC:ClY complexes and $SCCl^+$:^-Y ion-pairs

SC:ClY, ClY =	ρ_{BCP}	$\nabla^2\rho_{BCP}$	H_{BCP}
ClCl	0.022	0.069	0.001
ClNC	0.020	0.066	0.001
ClOH	0.021	0.069	0.001
ClCN	0.011	0.041	0.002
ClCCH	0.009	0.035	0.002
$ClNH_2$	0.010	0.036	0.002
$SCCl^+$:^-Y, Y=			
F	0.259	-0.394	-0.255
Cl	0.251	-0.385	-0.235
NC	0.266	-0.452	-0.266

Table S7. Components of ^{1x}J(C-Cl) for SC:ClY complexes and transition structures, ^{1}J(C-Cl) and ^{1x}J(Cl-A) for SCCl^{+}:$^{-}$Y ion-pairs, and ^{1}J(Cl-A) for monomers ClF, ClCl, and ClNC

	SC:ClY complexes				
ClY =	PSO	DSO	FC	SD	^{1x}J(C-Cl)
ClCl	0.2	0.0	62.7	-0.3	62.6
ClNC	0.1	0.0	60.2	-0.2	60.2
ClOH	0.2	0.0	56.2	-0.2	56.1
ClCN	0.1	0.0	28.2	0.0	28.3
ClCCH	0.1	0.0	21.6	0.0	21.7
ClNH2	0.1	0.0	17.8	0.0	18.0
	SCCl+:-Y				
Y =					^{1}J(C-Cl)
F	-13.3	0.1	-62.2	-1.7	-77.1
Cl	-10.4	0.1	-51.8	0.3	-61.9
NC	-14.0	0.1	-77.1	-2.1	-93.1
					^{1x}J(Cl-A)
F	24.0	0.1	422.1	3.2	449.3
Cl	12.0	0.0	34.9	3.9	50.7
NC	-0.6	0.0	-47.9	-0.1	-48.6
	TS				^{1x}J(C-Cl)
ClCl TS	-3.0	0.0	144.3	-0.8	140.5
ClNC TS	-5.4	0.1	142.1	1.7	138.4
Monomers					^{1}J(Cl-A)
ClF	642.2	0.1	-99.3	255.4	798.4
ClCl	78.5	0.0	-9.8	30.9	99.6
ClNC	-5.2	0.0	41.2	-2.0	34.0

Table S8. Components of ^1J(S-C) for complexes, ion-pairs, transition structures, and the SC monomer

SC:ClY complexes

ClY	PSO	DSO	FC	SD	^1J(S-C)
ClNC	-14.8	0.0	-24.8	4.0	-35.6
ClCl	-15.0	0.0	-25.6	3.8	-36.7
ClCN	-15.3	0.0	-25.1	3.7	-36.7
CLOH	-15.2	0.0	-25.7	3.7	-37.1
ClCCH	-15.7	0.0	-25.4	3.5	-37.5
ClNH2	-15.8	0.0	-26.1	3.5	-38.4
Ion-pairs					
ClF	-5.0	0.0	-30.7	4.7	-31.0
ClNC	-2.4	0.0	-28.3	5.8	-24.9
ClCl	-6.0	0.0	-36.9	3.0	-39.8
TS					
ClNC	-10.1	0.0	-31.7	1.1	-40.8
ClCl	-11.9	0.0	-32.6	4.7	-39.8
CS Monomer	-16.1	0.0	-26.5	3.3	-39.3
